# Reply: Her2 (ErbB2) receptors, a potential therapeutic target in squamous cell carcinoma of oesophagus?

**DOI:** 10.1038/sj.bjc.6603081

**Published:** 2006-04-18

**Authors:** J-P Metges, L Gibault, V Conan-Charlet, P Lozac'H, M Robaszkiewicz, C Bessaguet, N Lagarde, A Volant

**Affiliations:** 1 Institute of Oncology, Morvan Hospital, 5 Avenue Foch, 29609 Brest Cedex, France; 2 Department of Anatomical Pathology, La Cavale Blanche Hospital, Boulevard Tanguy Prigent, 29609 Brest Cedex, France; 3 General Surgery, La Cavale Blanche Hospital, Boulevard Tanguy Prigent, 29609 Brest Cedex, France; 4 Hepato-Gastroenterology, La Cavale Blanche Hospital, Boulevard Tanguy Prigent, 29609 Brest Cedex, France; 5 Biostatistics and Cancer Register Unit, Hepato-Gastroenterology, La Cavale Blanche Hospital, Boulevard Tanguy Prigent, 29609 Brest Cedex, France

**Sir**,

At first, we would like to thank you for giving us the opportunity to reply to this letter. It may be worth reminding that our study was aimed at gaining insight, from only immunohistochemical data, into the potential impact of several targets such as EGFR, Her 2, p53, VEGF and ckit in a large and homogenous series of patients (107) with oesophageal squamous cell carcinoma. Our experiments showed a potential impact of EGFR status, and HER2 was found to be overexpressed by only 2.8% of the tumours (Gibault *et al*, 2005).

Before replying to the letter by Khan, it sounds us worth (i) recalling that there is a consensus among the scientific community about the difficulty of comparing data relative to the assessment of angiogenesis by immunohistochemistry (Vermeulen *et al*, 2002) and (ii) indicating that our bibliography section did not refer to the two very interesting and recent papers by Mimura *et al* (2005a), 2005b) only because our manuscript had been submitted and accepted before the publication of both articles.

The remarks by Khan and co-workers are focused on the low rate reported by our team and on the potential role of HER2 in squamous cell carcinoma of the oesophagus. We totally agree with them about the fact that several analyses of HER2 carried out by immunohistochemistry or other methods (Shiga *et al*, 1993; Hardwick *et al*, 1997; Tanaka *et al*, 1997; Friess *et al*, 1999; Wang *et al*, 1999; Akatmatsu *et al*, 2003; Miyazono *et al*, 2004; Mimura *et al*, 2005a, 2005b; Sato *et al*, 2005; Sunpaweravong *et al*, 2005) have all found rates of overexpression greater than 2.8%. To our opinion, the differences observed between these different studies have several origins: the first of them, as clearly said by Khan and co-workers is the high heterogeneity in the methodologies used in the collection of studies cited by these authors. For example, Friess studied adenocarcinoma and squamous cell carcinoma and used an antibody and a semiquantitative procedure both different from ours (Friess *et al*, 1999). Among the available methods, the Dako Herceptest for immunohistochemical assay is considered as the most reliable one for the detection of a positive HER-2 status; this consideration drove us to use it and evaluate the staining as described by Penault-Llorca *et al* (2002). Concerning Mimura and co-workers, in addition to immunochemistry they performed a Her2 gene amplification with the aim to optimise their search for HER2 expression. However, by a careful reading of their paper in *Clinical Cancer Research* (Mimura *et al*, 2005a) one learns that, after the study of squamous cell lines, they analysed Her-2 expression in freshly isolated tumours (primary tumour and malignant pleural infusion from two patients; both samples were found to show no staining in Herceptest despite Her2 expression in flow cytometric analysis. In the second study (Mimura *et al*, 2005b) published in the *British Journal of Cancer*, they evaluated only HER2 in a homogenous series (66 cases) of squamous cell carcinoma of the oesophagus treated by surgery; one should note that, despite the lower number of cases under study, these investigations and ours can be compared. Among their cohort of patients, only three of them denoted (3+) were identified by immunochemistry as being positive; six others, denoted (2+), were considered as positive cases. When they compared the HER2 status between primary and metastatic lymph nodes, they clearly found a strong correlation, but only in the three HER2 (3+) (4.5% of the cases). FISH analysis revealed a gene amplification in the three strongly positive cases, that is (3+), in three out of the six samples denoted (2+) and in one out of the 11 weak positive patients termed (1+). To our knowledge, this study is the only report describing HER2 status in oesophageal neoplasms from results of the two FDA-approved tests. The finding that one patient with herceptest 1+ was positive for gene amplification suggests an underestimation of the rate of HER2-positive cases by this test.

Ongoing experiments within our laboratory are focused on a FISH analysis of our cohort of 126 patients. It should allow us to get data comparable with those reported by Mimura about 66 cases.

As we also wonder about the impact of cultural habits, ethnic differences between populations of the different continents, it would be also worth carrying out an intercomparison of HER2 and EGFR data in cohorts of patients from geographical areas where the incidence of squamous cell carcinoma is high (Japan, China, France or other countries) obtained by strictly using the same methodology to eventually highlight a difference of status in relation with the origin of patients and their food habits. Her-2 status is generally determined by FISH and immunohistochemistry. To our opinion, the serum HER2 is worth being analysed. Indeed, we reported at the ASCO GI 2006 symposium preliminary results about HER2, EGF, P53, VEGF and IL6 analysed by ELISA in the serum of patients treated by radiochemotherapy for oesophageal squamous cell carcinoma: pretherapeutic serum HER2 and serum EGF levels seemed to be strongly correlated (*P*=0.017). Moreover, serum HER2 levels suggested an association with progression (*P*=0.059) and metastatic status (*P*=0.006); but only pretherapeutic serum EGF levels were associated with overall survival (*P*=0.046) (Metges *et al*, 2006).

In conclusion, we think that HER2 is a potentially interesting target in oesophageal neoplasms. According to Mimura, Herceptest 3+ patients are the best candidates for anti-Her2 immune targeting; this subgroup of patients represents a relatively low proportion of the total population of oesophageal squamous cell carcinoma (Mimura *et al*, 2005b). However, further investigations needed to confirm these data. Prospective and homogenous series of patients required to analyse the potential interest of HER2 status by immunochemistry, serum analysis and gene amplification in the clinical course of patients with oesophageal squamous cell carcinoma.
